# Comparison of the complications of passive drainage and active suction drainage after pancreatectomy: A meta-analysis

**DOI:** 10.3389/fsurg.2023.1122558

**Published:** 2023-04-20

**Authors:** Zhou Xinyang, Lei Taoying, Lan Xuli, Chen Jionghuang, Zhong Framing

**Affiliations:** ^1^ Department of General Surgery, Wuyi First People's Hospital, Wuyi, China; ^2^ Department of General Surgery, Sir Run Run Shaw Hospital, The Affiliated Hospital of the Medical College, ZheJiang University, Hangzhou, China

**Keywords:** pancreatectomy, abdominal drainage, pancreatic fistula, meta-analysis, complications

## Abstract

**Objective:**

This study aimed to compare the effect of passive drainage and active suction drainage on complications after pancreatectomy.

**Methods:**

The databases were searched and covered in this study on the comparison of passive and active suction drainage after pancreatectomy from the database establishment to Feb. 2023. A meta-analysis was conducted with the RevMan5.3 software.

**Results:**

On the whole, 1,903 cases were included in eight studies, including 994 cases in the passive drainage group, 909 in the active suction drainage group, 1,224 in the pancreaticoduodenectomy group, as well as 679 in the distal pancreatectomy group. No statistically significant difference was identified between the two groups in the incidence of total complications, the rate of abdominal hemorrhage, the rate of abdominal effusion, the death rate and the length of stay after pancreatectomy (all *P* > 0.05), whereas the difference in the incidence of pancreatic fistula after distal pancreatectomy between the two groups was of statistical significance (OR = 3.35, 95% CI = 1.12−10.07, *P* = 0.03). No significant difference was reported in pancreatic fistula between the two groups after pancreaticoduodenectomy.

**Conclusion:**

After distal pancreatectomy, active suction drainage might down-regulate the incidence of postoperative pancreatic fistula.

## 1. Introduction

As medical imaging technology has been leaping forward, increasing pancreatic tumors can be detected at an early stage. Surgery refers to the first choice for treating pancreatic tumor, and it primarily fell to pancreaticoduodenectomy (PD) and distal pancreatectomy (DP) (1, 2). PD and DP were first completed and reported by German surgeon Billroth and Kausch in 1884 and 1909, respectively (3). Over the recent decades, as surgical technique and postoperative management have been advancing, the mortality after pancreatectomy has dropped significantly, whereas the incidence of postoperative complications remains high (4). In general, common complications after pancreatectomy consist of postoperative pancreatic fistula (POPF), abdominal hemorrhage, ascites, lung infection and others, in which pancreatic fistula is the most common and severe complication and may cause abdominal hemorrhage and a significant increase in mortality. For decades, reduce the incidence of pancreatic fistula, some treatment techniques (e.g., somatostatin, invagination anastomosis, mucosal anastomosis with catheter, pancreatic stent implantation) have been progressively employed (5–8).

Abdominal drainage tube has been recognized as another method to reduce the severity of postoperative pancreatic fistula and its relevant complications over the past few years, whereas it has once become a controversial topic. In 1992, Jeekel et al. reported that 22 cases who did not undergo an abdominal drainage tube after Whipple surgery achieved a good postoperative recovery. In the past three decades, considerable researchers have completed a number of randomized controlled studies to determine the significance of preventive drainage, whereas the research results have been significantly different (9, 10). For instance, a prospective randomized study by Van Buren et al. in 2013 reported that the absence of an abdominal drainage tube after PD increased the postoperative mortality of cases from 3% to 12%, thereby directly causing the early termination of the study (9). Accordingly, routine placement of abdominal drainage is still necessary after pancreatectomy at this stage, especially for cases with a higher risk of postoperative pancreatic fistula (11).

Abdominal drainage tubes can fall to passive drainage (PAD) and active suction drainage (ASD) by complying with different drainage methods. PAD makes the liquid flow out by gravity, while ASD can suck the liquid out through negative pressure suction (12). The existing choice of drainage mode after pancreatectomy remains controversial (13). This meta-analysis aimed to compare the effects of passive drainage and active negative pressure drainage on postoperative complications after pancreatectomy.

## 2. Data and methods

### 2.1. Search strategy

PRISMA guidelines were strictly followed during the study (14). All studies (e.g., PubMed, EMBASE, Cochrane Library, CNKI, CBM and Wanfang) were searched. The publication time of the study was from the establishment of the database to Feb 2023. English search terms included: Pancreatectomy, Pancreaticoduodenectomy, Distal Pancreatectomy, Postoperative pancreas, Passive drainage, active drainage. Chinese search terms included: pancreatectomy, pancreaticoduodenectomy, distal pancreatectomy, pancreatic surgery, passive drainage, and active drainage. The combination of subject word and free word retrieval was used for searching the study. The search was completed independently by two researchers.

### 2.2. Inclusion and exclusion criteria

Inclusion criteria: (1) Domestic and foreign published studies on comparison between passive drainage and active negative pressure drainage after pancreatectomy. (2) Complete data. (3) Prospective randomized controlled study or retrospective cohort study. (4) The number of samples is sufficient, with more than 30 cases in each group.

Exclusion criteria: (1) The study with only abstracts but without full text. (2) The study in which cases are not grouped. (3) The study does not provide complete data. (4) Repeated published study. (5) Review or meta-analysis, etc.

### 2.3. Literature screening and data extraction

Two researchers (XY-Z and TY-L) independently reviewed the titles and abstracts of all relevant articles. Then evaluate the full text, to prove whether they meet the eligibility criteria. In accordance with the inclusion criteria and exclusion criteria, the first literature screening was conducted by reading the title and abstract, the studies that did not meet the inclusion criteria were excluded, and then the second screening was conducted by reading the full text. The screening was performed by two researchers independently and cross checked to resolve differences through discussion. Data were extracted from the final included studies, which included (1) general information (e.g., title, author, publication date and country); (2) the basic characteristics of the research object, the sample size, age and gender of the grouping; (3) the postoperative complications, mortality and other outcome indicators concerned by the respective study.

### 2.4. Quality assessment

The quality of the included studies was assessed by complying with to RCTs' quality assessment criteria recommended by Cochrane Handbook 5.2. The risk of bias assessment chart was generated with the Cochrane Collaboration Revman 5.3 software. The risk of bias assessment categories included (1) random sequence generation; (2) allocation concealment; (3) blinding of participants; (4) blinding of outcome assessors; (5) completeness of outcome data; (6) selective outcome reporting; (7) other biases. The assessments for the respective item would be graded as “yes” (low risk), “no” (high risk), and “unclear” (lack of relevant information or uncertain bias) to assess several risks of bias. For case-control studies, the Newcastle-Ottawa Scale (NOS) was employed to assess the included studies’ quality, which contained three aspects, i.e., case selection, comparability and exposure. The highest quality of studies was 9 stars, and the lowest was 0 star.

Two researchers independently assessed the quality of the study and performed cross-check, and the discrepancies were resolved *via* discussion.

### 2.5. Statistical analysis

The fixed effect model or the random effect model was selected according to heterogeneity, and then odd ratio (OR) and 95% confidence interval (IC) were calculated as the overall assessment indexes. The sensitivity analysis was performed from the perspectives of the single factor analysis (excluding uncontrolled confounding factors) and the ransformation model. If the results did not change significantly, it was indicated that the research results were reliable. If the median, maximum and minimum values of samples were applied in the included studies, the method proposed by Hozo et al. (15) was adopted to estimate the mean and standard deviation of samples, respectively. *P* < 0.05 was statistically significant. The funnel plot was employed to assess the potential publication bias. When less than 10 studies were included, the test efficiency was extremely low, so the publication bias test was not required to be performed (16).

Given the differences between PD and DP, the subgroup analysis was conducted to compare the effects of passive drainage and active negative pressure drainage on postoperative complications of PD and DP.

## 3. Results

### 3.1. Results of search

**Figure 1:**
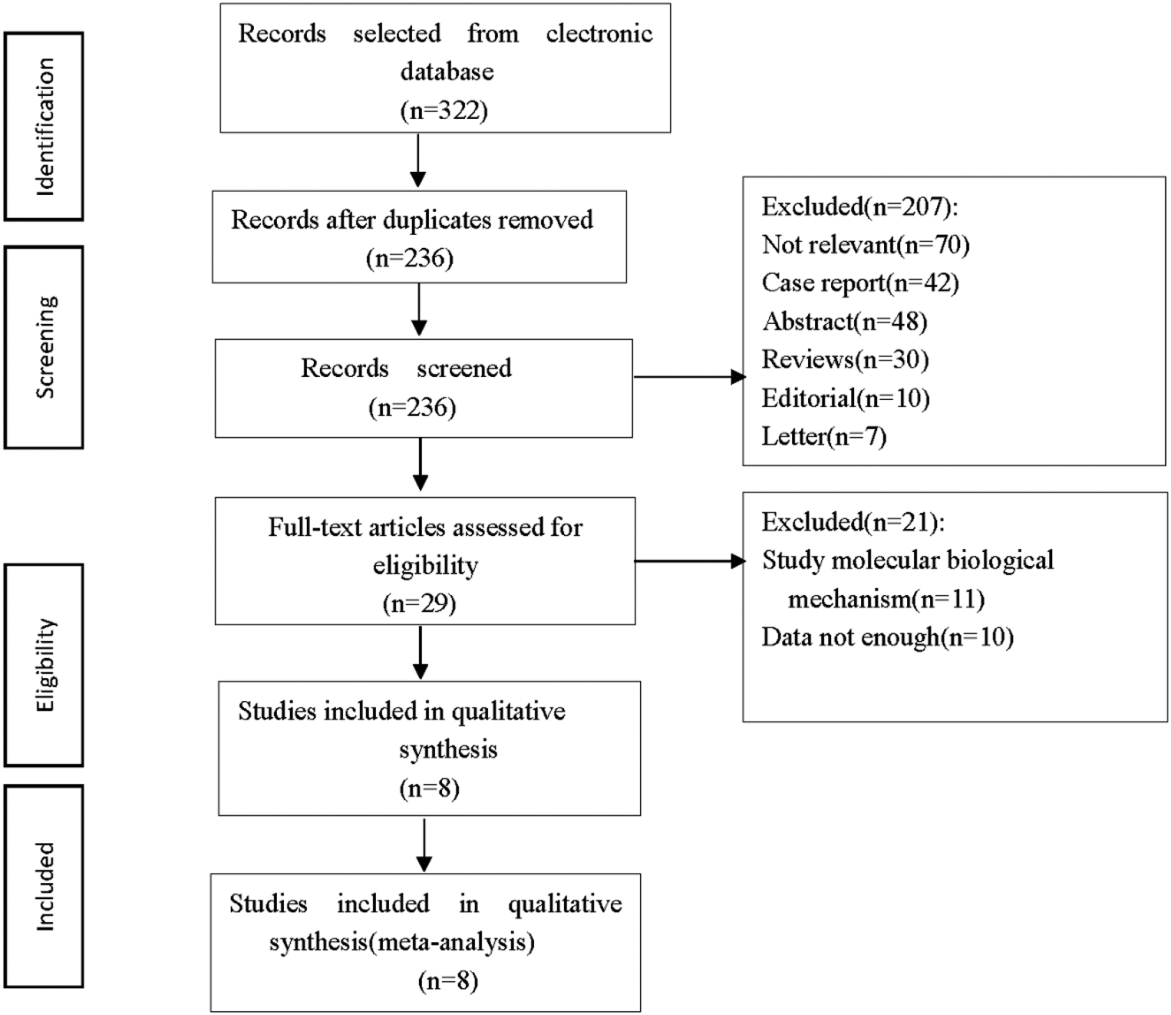
Literature screening and results.

The study screening is shown in Figure 1. Lastly, 8 studies met the inclusion criteria, including 3 prospective studies and 5 retrospective studies, which were from France, Czech Republic, China, Italy, the United States and Japan, with a total of 1,903 cases, including 994 cases in the PAD group and 909 cases in the ASD group.The general information of the included studies is listed in Table 1.

**Table 1:** The general information of the included studies.

Ref.	Study	Year	Region	Age (median)	Gender (M/F)	Cases	
						PAD group	ASD group
(17)	Aumont et al.	2017	France	66.2	1.21:1	132	65
(18)	Čečka et al.	2018	Czech Republic	63.9	0.96:1	111	111
(19)	Dokmak et al.	2019	France	56	0.62:1	79	102
(20)	Jiang et al.	2016	China	59.6	2.81:1	78	82
(24)	Marchegiani et al.	2018	Italy	62.9	1.09:1	189	131
(25)	Schmidt et al.	2009	America	58	1.25:1	241	269
(26)	Vanbrugghe et al.	2018	France	58	0.72:1	112	92
(27)	Yui et al.	2014	Japan	65.5	1.02:1	52	57

PAD, passive drains; ASD, active suction drainage.

### 3.2. Quality assessment of included studies

**Figure 2:**
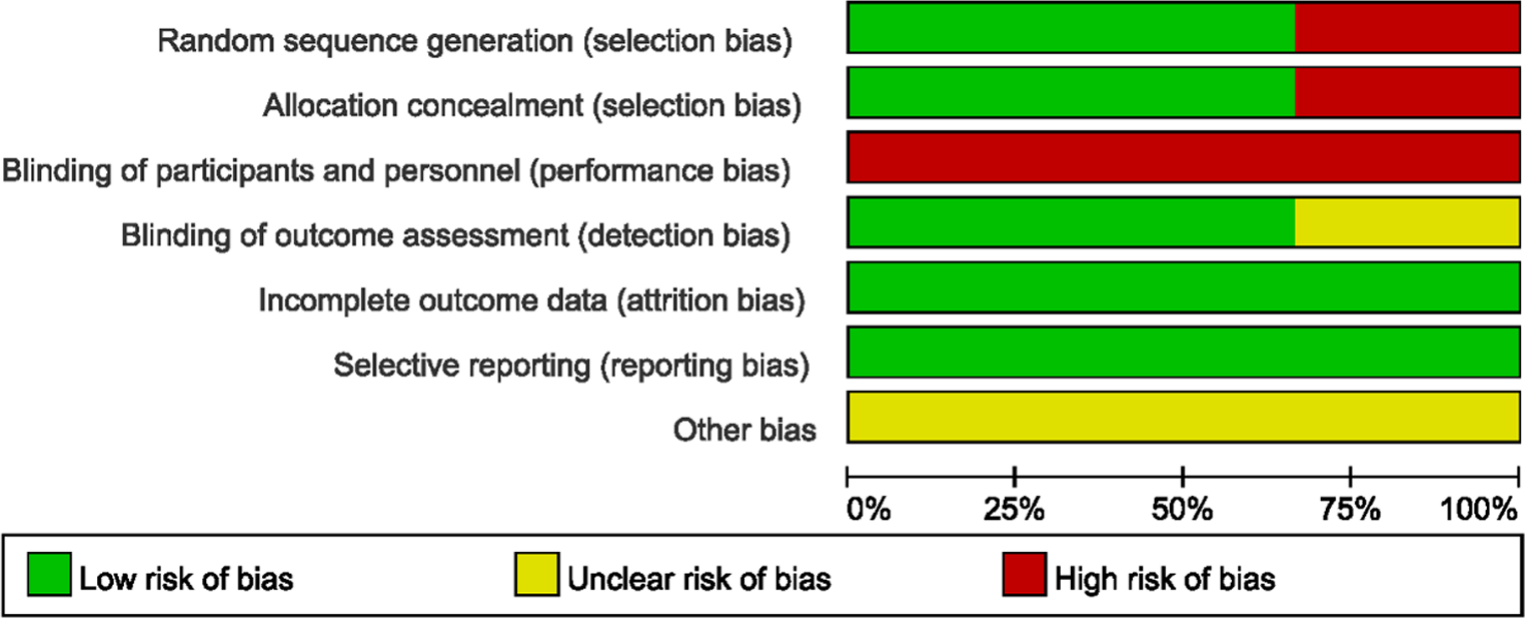
Bias risk assessment chart of the prospective randomized controlled studies.

**Figure 3:**
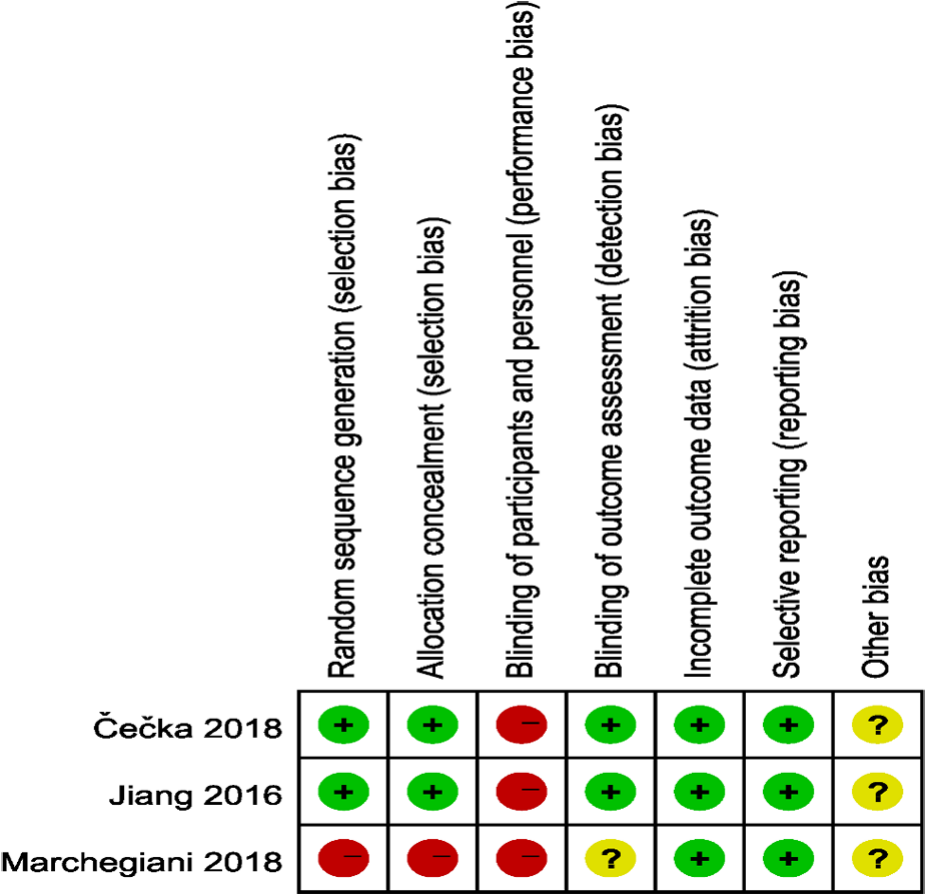
Bias risk assessment chart of the prospective randomized controlled studies.

The study quality assessment of retrospective cohort study and prospective randomized controlled study are listed in Table 2 and Figures 2, 3 respectively.

**Table 2:** Literature quality assessment of the retrospective cohort study (Newcastle-Ottawa scale).

Study	Selection				Comparability	Exposure			Total Quality score
	①	②	③	④	⑤	⑥	⑦	⑧	
Aumont et al.	*	*	–	*	–	*	*	–	5
Dokmak et al.	*	*	–	*	–	*	*	–	5
Schmidt et al.	*	*	–	*	–	*	–	–	4
Vanbrugghe et al.	*	*	–	*	–	*	–	–	4
Yui et al.	*	*	–	*	–	*	*	–	5

① Is the case definition adequate. ② Representativeness of the cases. ③ Selection of Control. ④ Definition of Controls. ⑤ Comparability of cases and controls on the basis of the design or analysis. ⑥ Ascertainment of exposure. ⑦ Same method of ascertainment for cases and controls. ⑧ Non-Responsate rate. – indicates a score of 0, * indicate a score of 1.

### 3.3. Results of meta-analysis

#### 3.3.1. Incidence of total postoperative complications

**Figure 4:**
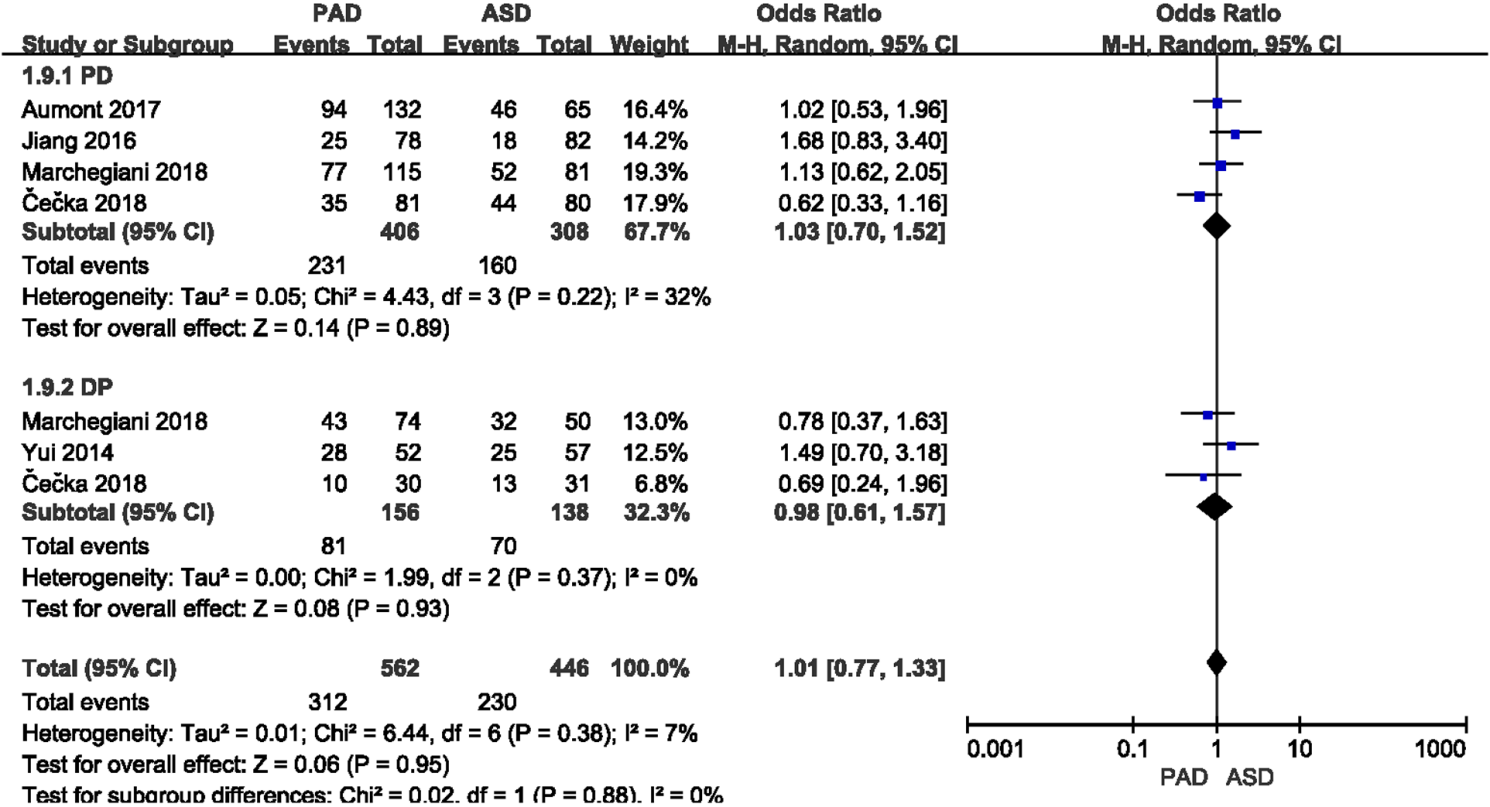
Results of the total postoperative complication rate in PAD and ASD groups.

Total postoperative complications were reported in five included studies (Figure 4). As indicated from the meta-analysis, no significant difference was identified in the incidence of postoperative complications between the two groups after PD (OR = 1.03, 95% CI = 0.70–1.52, *P* = 0.89) and DP (OR = 0.98, 95% CI = 0.61–1.57, *P* = 0.93).

#### 3.3.2. Incidence of postoperative pancreatic fistula

**Figure 5:**
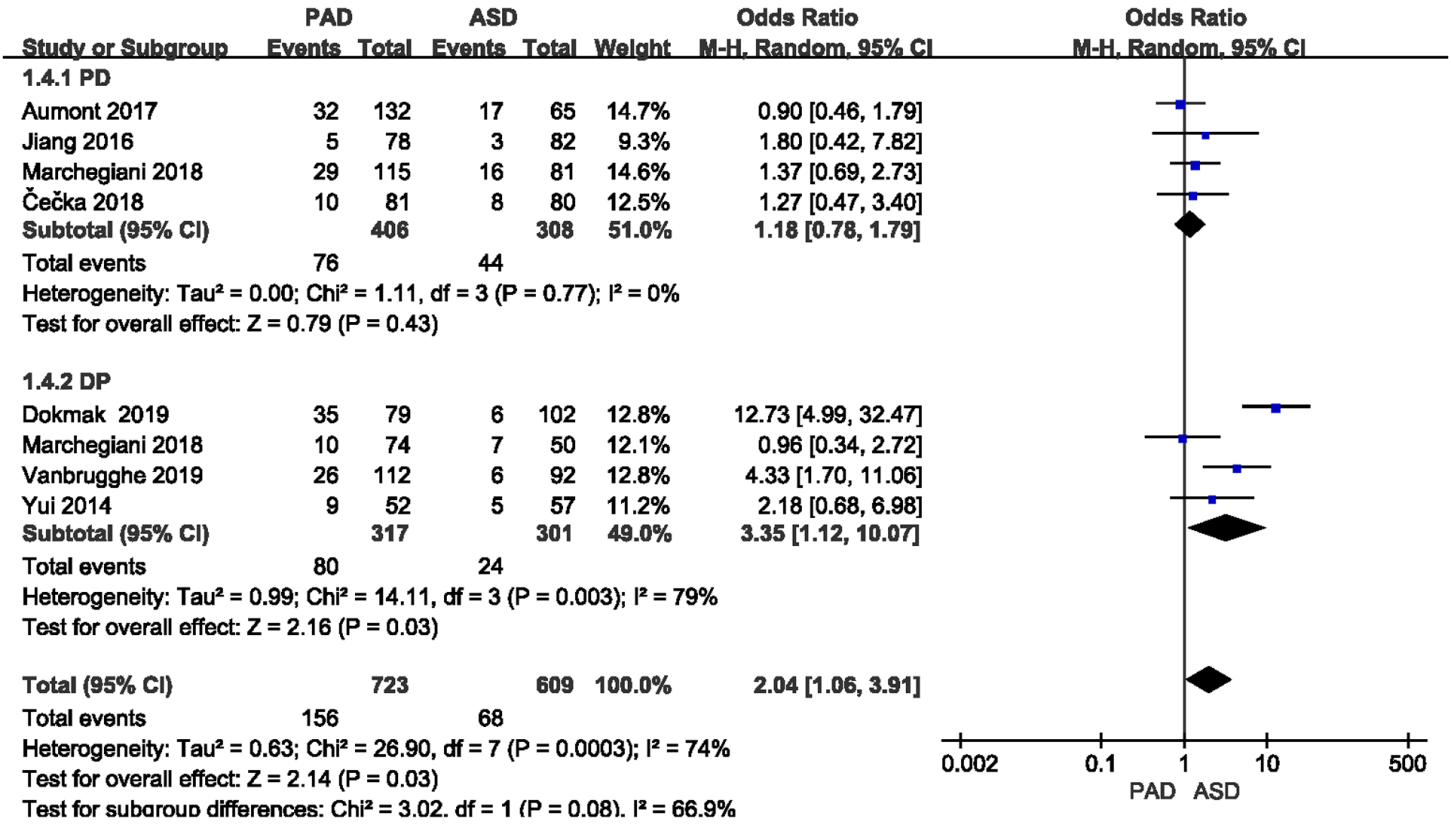
Results of the POPF rate in PAD and ASD groups.

POPF was reported in all eight included studies (Figure 5). The classification of the POPF definition has been updated over the past few years (17, 18). The 2005 edition of the International Study Group of Pancreatic Fistula (ISGPF) classified pancreatic fistula into three grades, i.e., A, B and C (28). The 2016 edition of ISGPF classified the A-grade pancreatic fistula as biochemical leakage, which did not pertain to pancreatic fistula (29), and the B-grade and C-grade pancreatic fistula were considered as pancreatic fistula. Given the significant heterogeneity (*I*^2^ = 79%) between the two groups after DP, the random effect model was applied. A significant difference was found in the incidence of pancreatic fistula after DP (or = 3.35, 95% CI = 1.12–10.07, *P* = 0.03), whereas no significant difference was reported in the incidence of pancreatic fistula after PD (or = 1.18, 95% CI = 0.78–1.79, *P* = 0.43).

#### 3.3.3. Incidence of postoperative abdominal bleeding

**Figure 6:**
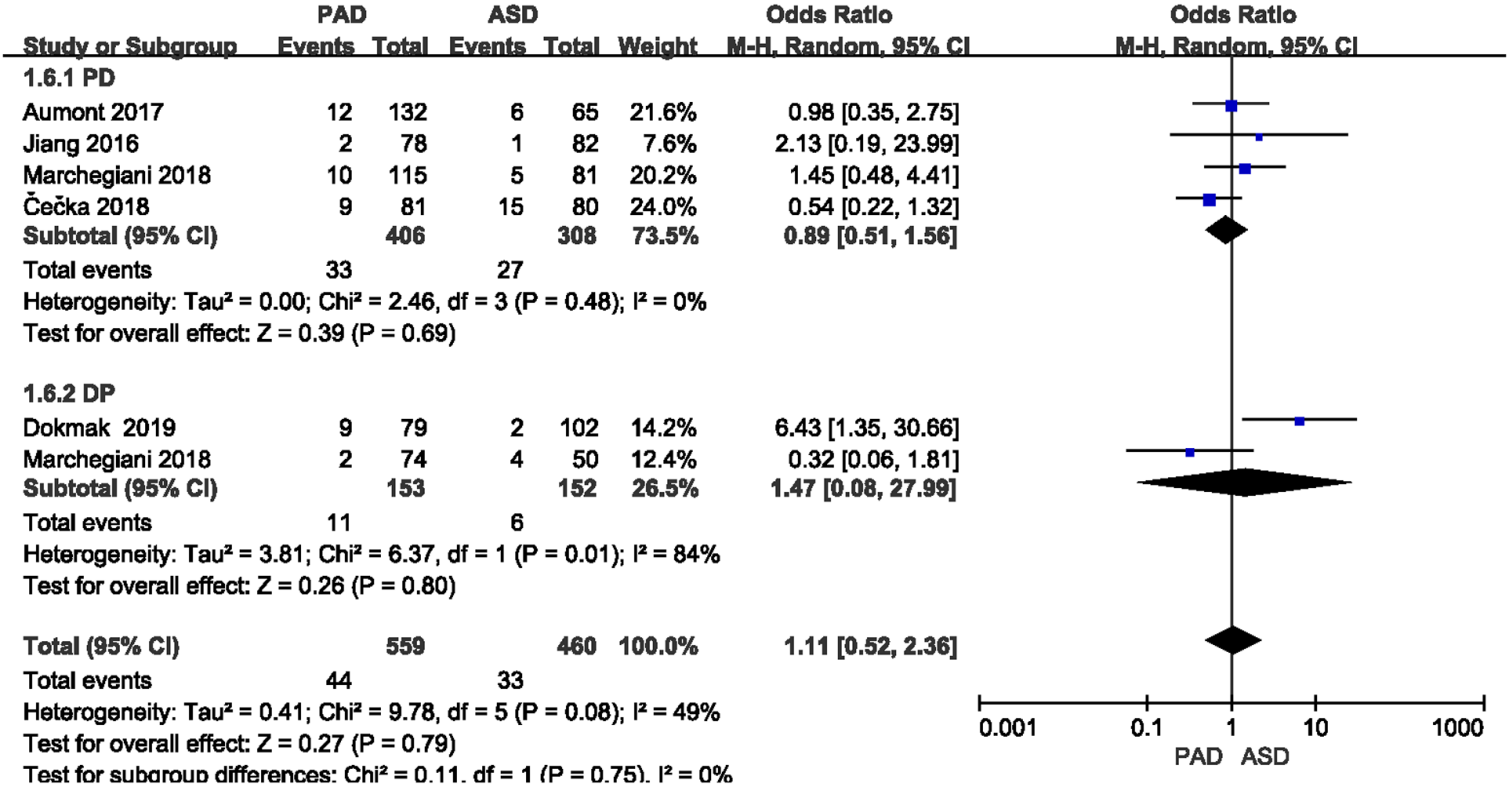
Results of the postoperative abdominal bleeding rate in PAD and ASD groups.

The incidence of postoperative abdominal bleeding was reported in five included studies (Figure 6). As suggested from the meta-analysis, no significant difference was identified in the incidence of postoperative abdominal bleeding between the two groups after PD and DP (OR = 0.89,95% CI = 0.51–1.56, *P* = 0.69) and (OR = 1.47,95% CI = 0.08–27.99, *P* = 0.80).

#### 3.3.4. Postoperative ascites rate

**Figure 7:**
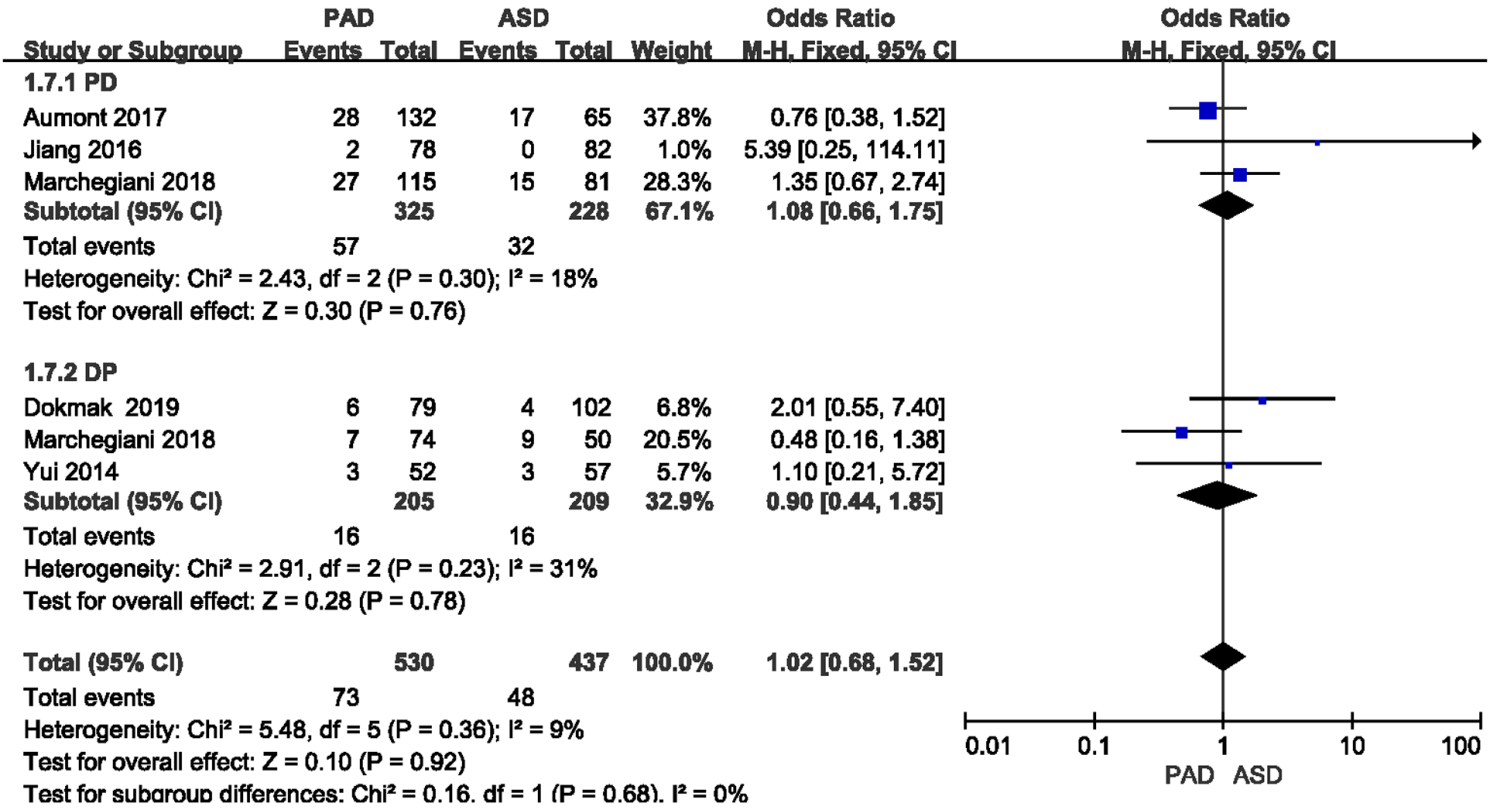
Results of the postoperative ascites rate in PAD and ASD groups.

The postoperative ascites rate was reported in five included studies (Figure 7). As revealed from the meta-analysis, no significant difference was reported in postoperative ascites rate between the two groups after PD and DP (OR = 1.08, 95% CI = 0.66–1.75, *P* = 0.76) and (OR = 0.90, 95% CI = 0.44–1.85, *P* = 0.78).

#### 3.3.5. Postoperative mortality

**Figure 8:**
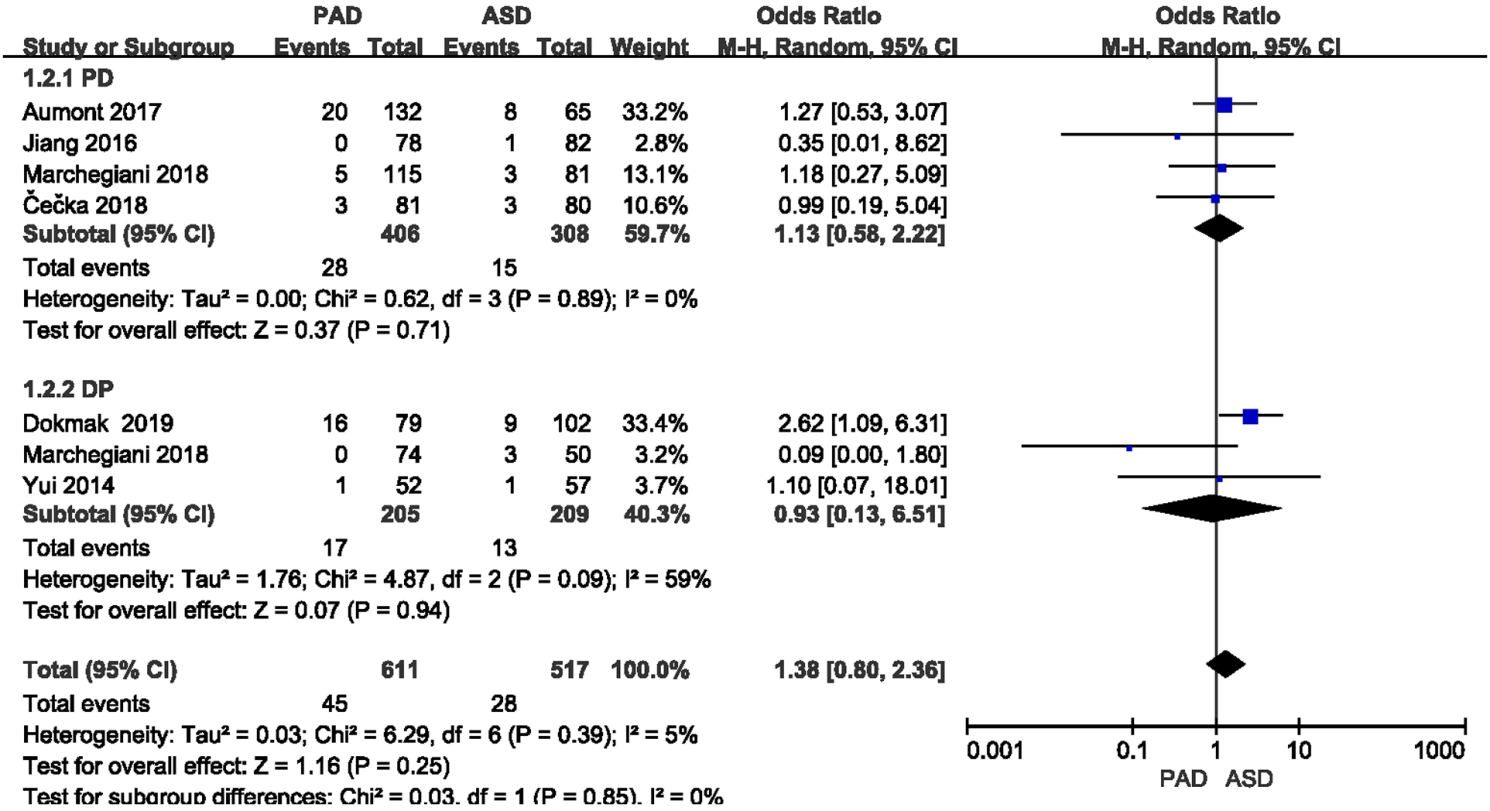
Results of the postoperative mortality rate in PAD and ASD groups.

The postoperative mortality was reported in six included studies (Figure 8). As indicated from the meta-analysis, no significant difference was identified in postoperative mortality between the two groups after PD and DP (OR = 1.13,95% CI = 0.58–2.22, *P* = 0.71) and (OR = 0.93, 95% CI = 0.13–6.51, *P* = 0.94).

#### 3.3.6. Length of hospitalization

**Figure 9:**
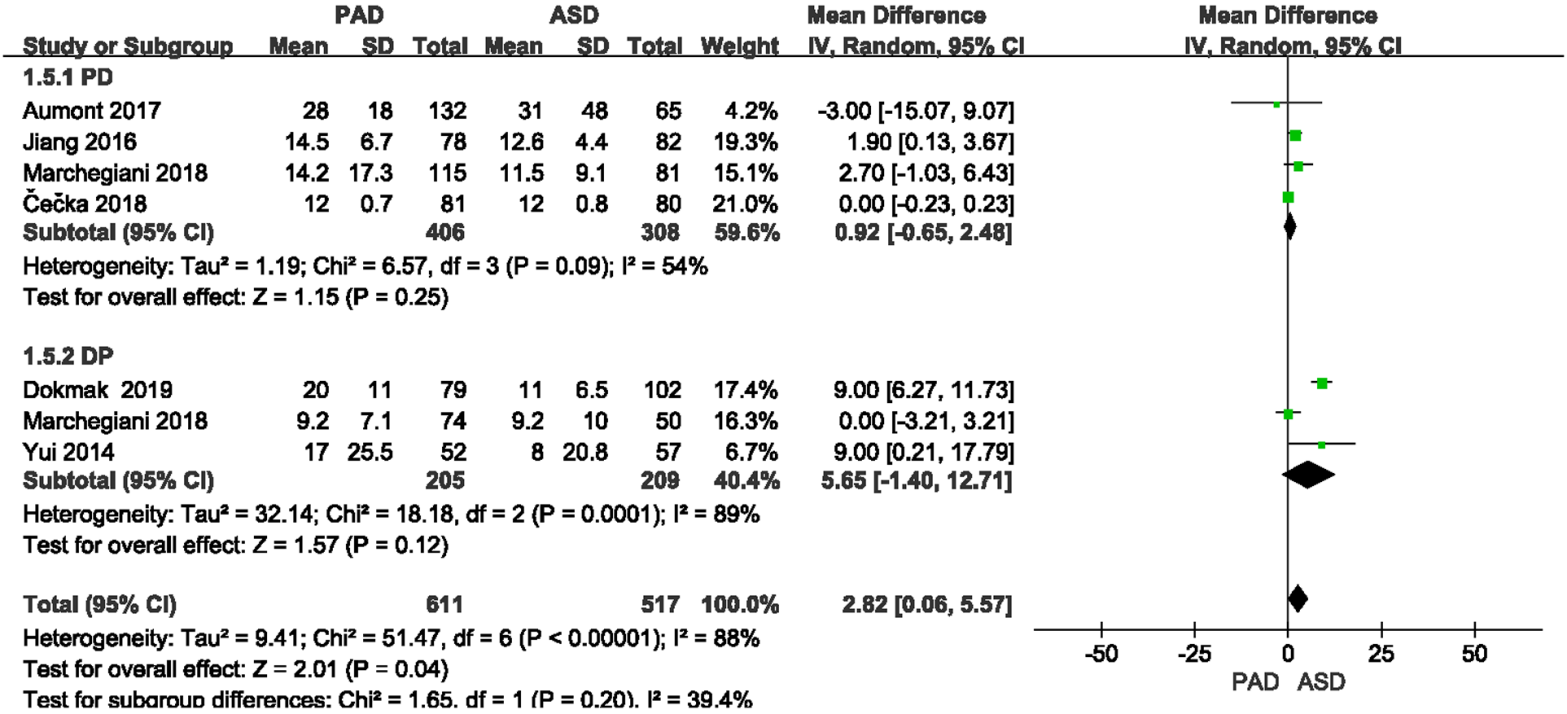
Results of the length of hospitalization in PAD and ASD groups.

The length of hospitalization was reported in six included studies (Figure 9). As suggested from the meta-analysis, no significant difference was reported in length of hospitalization between the two groups after PD and DP (OR = 0.92, 95% CI = −0.65–2.48, *P* = 0.25) and (OR = 5.65, 95% CI = −1.4–12.71, *P* = 0.12).

### 3.4. Sensitivity analysis

As reported from the heterogeneity test, the incidence of postoperative pancreatic fistula, postoperative bleeding rate, mortality and other heterogeneity was high, so the sensitivity analysis was conducted by one-by-one elimination method. After the included studies were excluded one by one, the above outcome indicators were basically the same, which demonstrated that the results of meta-analysis were stable and reliable.

## 4. Discussion

In the existing clinical work, most surgeons still prefer using preventive abdominal drainage after pancreatectomy. The primary purpose refers to draining the residual effusion and hematocele during the surgery, reducing the potential secondary abdominal infection; to observing the nature and quantity of postoperative abdominal drainage fluid and testing the drainage fluid, as an attempt to diagnose the pancreatic fistula, bile leakage, intestinal leakage and abdominal bleeding earlier; to performing better drainage of complications (e.g., pancreatic fistula, bile leakage and intestinal leakage), as an attempt to avoid corrosion of adjacent tissues, secondary bleeding and infection. Active negative pressure drainage exerts a noticeable drainage effect by using the negative pressure suction device, and negative pressure can reduce the risk of bacterial retrograde infection as well. Passive drainage is weaker than active negative pressure drainage, and it is readily affected by body position change, whereas it causes slight damage to the tissue (20). In clinical practice, the types of abdominal drainage tubes placed after pancreatic surgery should be analyzed according to patients' conditions (preoperative disease complications, perioperative risk assessment, coagulation conditions, etc.) and surgical conditions (pancreatic texture, pancreatic duct diameter, surgical time, amount of blood loss, surgical proficiency, and surgical type). Postoperative passive drainage is often selected if the patient has a low risk of postoperative pancreatic fistula, such as hard pancreas texture, thick pancreatic duct, short operation time and less bleeding (21–23). Both drainage methods have been extensively used in different nations. Passive drainage is widely used in European pancreatic centers, while doctors in the United States and Asia are inclined to use active negative pressure drainage (24). In clinical practice, the choice of drainage mode after pancreatectomy remains controversial, and the relevant research results are also inconsistent. Thus, this meta-analysis has specific guiding significance for clinical work.

In this study, a meta-analysis of eight studies meeting the inclusion and exclusion criteria was conducted. The results showed that the incidence of POPF was lower in active negative pressure drainage after DP than in passive drainage, and no significant difference was identified in the incidence of POPF between active negative pressure drainage and passive drainage after PD. This study also suggested no statistical difference in total postoperative complications, abdominal bleeding rate, ascites rate, postoperative mortality rate and length of hospitalization between the two groups.

The lower rate of POPF in the active negative pressure drainage group after DP might be mainly because it could more fully discharge abnormal accumulated fluid in abdominal cavity (e.g., digestive juice attributed to anastomotic leakage or hematocele in abdominal cavity), which can avoid or reduce pancreatic fistula caused by infection and corrosion of surrounding pancreatic tissue, as well as effectively avoid or reduce the development from mild pancreatic fistula to more severe pancreatic fistula. Besides, some researchers have a concern that negative pressure will suck pancreatic juice through anastomosis or suture, thereby leading to or aggravating postoperative pancreatic fistula and bleeding rate (25). However, the meta-analysis results here showed that the above risks did not occur when lower suction was used.

Though the data in this meta-study were carefully checked and analyzed, some limitations remained (Supplementary Table S1). First, the number of studies included was limited, and there were too many retrospective studies. Second, there were differences in the medical conditions and technology of hospitals in different countries, which might also affect the results of the analysis. Third, there were some differences in the updating of the relevant definitions and classification, and the research data statistics might exert a specific impact on the results. Lastly, the assessment results of the included studies would also bring related heterogeneity. Accordingly, the data of multi-center, large sample and prospective randomized controlled study are still required in the future to support the assessment of the curative effect of the above two drainage methods after pancreatectomy.

As indicated from this study, the incidence of postoperative pancreatic fistula might be reduced by active negative pressure drainage after DP, whereas no significant difference was identified in the incidence of postoperative pancreatic fistula after PD. The factors of pancreatic fistula after pancreatectomy are very complicated, and the relationship between pancreatic fistula and drainage mode remains unclear. Thus, the authors also have doubts about the results of this meta-analysis. Moreover, potential heterogeneity existed in the clinical and methodological aspects of the present meta-analysis, which should be carefully explained and further verified by using a large sample and high-quality randomized controlled studies.
